# Left atrial cardiac Tamponade complicating radical nephrectomy – case report

**DOI:** 10.1186/s13019-020-01190-3

**Published:** 2020-07-08

**Authors:** Dominic Thompson, Ihab Ali, Michael Gilbert, Afzal Zaidi

**Affiliations:** 1 Morriston Hospital, Swansea, United Kingdom; 2 Ain Shams University, Cairo, Egypt

**Keywords:** Cardiac tamponade, Left atrial tamponade, Radical nephrectomy, Pericardial effusion

## Abstract

**Background:**

*We present an unusual case of pericardial tamponade occurring subsequent to a radical right nephrectomy performed entirely through a laparotomy.*

**Case presentation:**

*A 43 year old gentleman who presented with large loculated posterior pericardial effusion compressing the left atrium, following a radical nephrectomy performed entirely through a laparotomy. He required an emergency sternotomy, pericardial and pleural drainage.*

**Conclusion:**

*Cardiac tamponade is an extremely rare complication of radical nephrectomy. However, any procedure that involves opening of the pericardium does carry a risk of bleeding and therefore cardiac tamponade, particularly in the context of post-operative full anticoagulation.*

## Background

We present an unusual case of pericardial tamponade occurring subsequent to a radical right nephrectomy performed entirely through a laparotomy.

## Case presentation

A 43 year old gentleman with no past medical history of note presented to his General Practitioner with 2 episodes of frank haematuria. An ultrasound scan was carried out which demonstrated a large mass in the right kidney. A follow-up contrast enhanced computerised tomography (CT) scan of the thorax, abdomen and pelvis was carried out which demonstrated a large right intrarenal tumour with extracapsular spread into the right adrenal gland and vascular invasion of the right renal vein and inferior vena cava (IVC). An echocardiogram demonstrated no invasion of the right atrium.

A right radical nephrectomy with exploration of the IVC was performed jointly by a urological and cardiothoracic surgeon. The operation was performed entirely via a laparotomy using an L-shaped anterior abdominal incision to facilitate mobilisation of the suprahepatic IVC. The diaphragm was divided radially from the xiphoid down to the IVC. Cross clamping of the IVC below the right atrium allowed removal of the tumour from within it, thus no sternotomy or cardiopulmonary bypass was used. At the end of the operation the diaphragm was repaired from below. The pericardium was opened in order to facilitate removal of the tumour and a pericardial drain was placed following the procedure. The operation was without complication, however 3 days post-operatively the patient developed a pulmonary embolism (PE) and was anticoagulated with treatment dose tinzaparin. The patient recovered well and was discharged on day 10 post-operatively.

On the 19th post-operative day the patient presented to his local emergency department with abdominal discomfort, nausea and bilious vomiting which had lasted 24 h. On examination there was mild abdominal tenderness and epigastric fullness. His observations were stable, ECG showed normal sinus rhythm and bloods demonstrated no clear abnormality other than a C-reactive protein (CRP) of 50 and an alkaline phosphatase (ALP) of 172. Following a period of observation, he was discharged home. The working diagnosis given at this time was viral gastroenteritis.

The following day the patient returned to the emergency department in cardio-respiratory failure. He had a tachycardia of 120**,** was severely agitated and was hypoxic with oxygen saturations of 82%**.** He was coughing up large amounts of blood stained frothy sputum. His blood tests demonstrated rising leucocyte count of 14 and CRP of 99, which increased overnight to a leucocyte count of 17.9, CRP 117 and of note a troponin of 478. The decision was made to intubate in order to facilitate a CT pulmonary angiogram (CTPA) as a recurrent PE was the initial working diagnosis. Following sedation he was profoundly bradycardic and hypotensive, necessitating rapidly increasing pharmacological cardiovascular support, and on insertion of the endotracheal tube a large volume of pulmonary oedema fluid was suctioned. The CTPA demonstrated no PE, however there was extensive pulmonary airspace shadowing in both lower lobes consistent with acute respiratory distress syndrome (ARDS), as well as a highly abnormal appearance to the left atrium with a smooth margined filling defect extending from the posterior wall anteriorly compressing the lumen. There was a 6 cm pericardial effusion seen. Furthermore, a transthoracic echocardiogram demonstrated a huge cystic lesion in the left atrium with a thick wall containing fluid and debris. These scan results were discussed with the regional cardiothoracic unit and an emergency transfer was performed.

The patient was taken straight to theatre and the transoesophageal echocardiogram confirmed a large (6.68 × 7.25 cm) loculated posterior pericardial effusion which at this point was entirely compressing the left atrium. An emergency sternotomy was performed, a large posterior loculated effusion was found which was made up of old venous blood, 450 ml was drained and a pericardial drain placed. The patient’s cardiac output immediately improved and norepinephrine requirement decreased. The pleura was opened bilaterally with straw coloured fluid drained 500 ml on the left and 300 ml on the right, drains were placed bilaterally.

He was extubated the following day, and biochemically improved significantly over the following 4 days with his leucocyte count completely normalising. Serial chest radiographs demonstrated significant improvement with reduction in pulmonary oedema, this was mirrored by his oxygen requirements completely reducing. The patient was discharged home and outpatient follow up demonstrated an excellent recovery.

## Discussion

This patient developed a massive posterior pericardial effusion and tamponade (19 days post-operatively). At his original surgery, the diaphragm was divided inferiorly and the pericardium entered, the diaphragm was closed from the inferior aspect. 3 days post-operatively he was fully anticoagulated following a PE.

We hypothesise that there was a small vessel in the diaphragm, which continued to bleed because of the full anticoagulation. This resulted in a massive collection of blood in the posterior pericardial space (oblique sinus) which caused complete compression of the left atrium manifesting as left atrial cardiac tamponade and consequent pulmonary oedema. To our knowledge this is the first reported incidence of this condition in this situation.

A review of the literature shows that there are no specific cases or studies found that demonstrated cardiac tamponade following radical nephrectomy. The most closely related case report is of cardiac tamponade which developed 7 years after a radical nephrectomy, related to metastatic spread of disease [3]. Locali et al. followed up 14 patients who had tumours, which were invading their inferior vena cava resected [1]. From this cohort, 2 patients developed cardiac tamponade at some point following tumour resection. One of these patients required an operation for pericardial drainage; however more specific details regarding these patients are not given. Osman et al. demonstrated an unusual case of cardiac tamponade in a patient who was 3 months post radical nephrectomy for renal cell carcinoma, who presented with a pericardial effusion and a ventricular septal tumour [2]. Yildiz et al. described cardiac tamponade, suggested to be due to treatment with Sunitinib causing a possible complication of cardiac toxicity [4]. However, this patient 1 year prior had a left radical and right partial nephrectomy carried out. Due to the timeline of presentation it is unlikely that these nephrectomies were related to the pericardial tamponade. As demonstrated above there are limited cases within the literature where pericardial effusion is demonstrated as a complication following a radical nephrectomy. This literature demonstrated metastatic disease as a common cause for cardiac tamponade in renal cell carcinoma.

## Conclusion

**Fig. 1:**
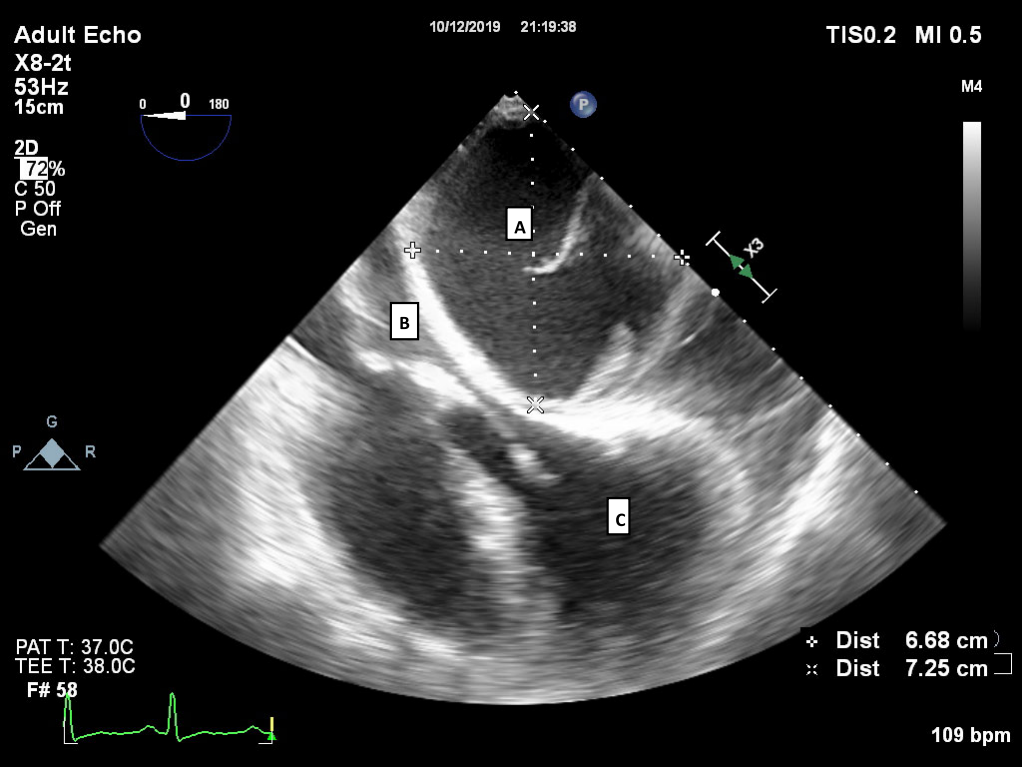
**a** Large Pericardial Collection. **b** Left atrium compressed to a slit. **c** Left Ventricle

Cardiac tamponade is an extremely rare complication of radical nephrectomy. However, any procedure that involves opening of the pericardium does carry a risk of bleeding and therefore cardiac tamponade, particularly in the context of post-operative full anticoagulation (Fig. 1).

## Data Availability

All supporting data has been provided.

## Abbreviations

CT: Computerised tomography
IVC: Inferior vena cava
PE: Pulmonary embolism
CRP: C-reactive protein
ALP: Alkaline phosphatase
CTPA: computerised tomography pulmonary angiogram
ARDS: Acute respiratory distress syndrome
